# Applying randomized control trial criteria to an ECPR cohort

**DOI:** 10.1016/j.resplu.2026.101269

**Published:** 2026-02-12

**Authors:** Yigal Helviz, Frederic Shmuel Zimmerman, Amir Orlev, Michael Glikson, David Shimony, Daniel Fink, Refael Strugo, Elad Asher, Tomer Kaplan, Reem Naimy, Ofer Merin, Phillip D. Levin

**Affiliations:** a Intensive Care Unit, Shaare Zedek Medical Center, Jerusalem, Israel; b Faculty of Medicine, Hebrew University of Jerusalem, Israel; c ECMO Service, Shaare Zedek Medical Center, Jerusalem, Israel; d Jesselson Integrated Heart Center, Shaare Zedek Medical Center, Jerusalem, Israel; e Cardiothoracic Surgery Department, Shaare Zedek Medical Center, Jerusalem, Israel; f Medical Division and Blood Bank Manager, Magen David Adom, Israel; g Patient Safety Department, Medical Division, Magen David Adom, Israel; h Medical Supervisor, Jerusalem Region, Magen David Adom, Israel; i Director General, Shaare Zedek Medical Center, Jerusalem, Israel

**Keywords:** Out of hospital cardiac arrest, OHCA, Extracorporeal membrane oxygenation, ECMO, ECMO cardiopulmonary resuscitation, ECPR

## Abstract

**Objective:**

To assess how inclusion criteria from major randomized controlled trials (RCTs) of extracorporeal cardiopulmonary resuscitation (ECPR) apply within a cannulated ECPR cohort and to benchmark observed outcomes against published trial results.

**Methods:**

We conducted a single-center retrospective cohort study at a 1,000-bed tertiary medical center of adults who underwent ECPR for out of hospital cardiac arrest. Inclusion criteria from the ARREST, PRAGUE, and INCEPTION trials were retrospectively applied. Analyses were restricted to cannulated patients, with survival evaluated overall and stratified by trial eligibility, and descriptively compared with published RCT outcomes.

**Results:**

Sixty-six patients underwent ECPR, including 25 (38%) with a non-shockable initial rhythm. Overall survival to hospital discharge was 14% (9/66; 95% CI 6.4–24.3%), with favorable neurological outcome in 5/9 survivors. Survival was numerically higher among patients meeting trial inclusion criteria, but differences were not statistically significant. Survival was comparable to PRAGUE and INCEPTION and lower than ARREST.

**Conclusions:**

Within this program level, cannulated ECPR cohort, RCT-derived inclusion criteria did not clearly distinguish survivors from non-survivors, and survival occurred among patients not meeting one or more trial eligibility thresholds. Rigid application of trial criteria at the point of cannulation may therefore exclude some patients with potential for meaningful recovery.

## Introduction

Out of hospital cardiac arrest (OHCA) carries poor survival despite advances in prehospital care and resuscitation systems.^1^, ^2^ Extracorporeal cardiopulmonary resuscitation (ECPR) using ExtraCorporeal Membrane Oxygenation (ECMO) has emerged as a potential strategy for select patients with refractory arrest; however, its effectiveness and optimal candidate selection remain uncertain.^3^, ^4^, ^5^ To date, three randomized controlled trials (RCTs) have evaluated ECPR in OHCA with divergent results. The single center ARREST trial demonstrated a marked survival benefit and was stopped early for efficacy,^6^ whereas the single-center PRAGUE trial, which employed earlier randomization, was terminated for futility regarding favorable neurological outcome.^7^ More recently, the multicenter INCEPTION trial, which included centers with variable ECPR experience, found no improvement in survival with favorable neurological outcome.^8^

These trials differed substantially in their inclusion criteria, timing of randomization, transport strategies, and system capabilities, factors that likely contributed to the heterogeneity of outcomes.

Although inclusion criteria are designed to ensure internal validity in RCTs, they are subsequently used to inform guideline recommendations, triage algorithms, and ECPR candidacy decisions.^9^ As such, understanding how these criteria map onto patients who ultimately undergo ECPR in clinical programs is important.

Given these differences, it remains unclear how the eligibility criteria used in ECPR trials correspond to the characteristics of patients who undergo ECPR within established programs. Examining how RCT inclusion criteria apply within a cannulated ECPR cohort may help contextualize trial findings and inform program level benchmarking. We hypothesized that ECPR randomized controlled trial inclusion criteria would identify a subgroup with higher survival within our cohort.

## Materials and methods

### Study setting and design

We conducted a retrospective cohort study at Shaare Zedek Medical Center (SZMC), a 1000 bed tertiary hospital and the largest medical center in Jerusalem. The study included all consecutive cases of extracorporeal cardiopulmonary resuscitation (ECPR) performed for out-of-hospital cardiac arrest (OHCA) between January 2018 and April 2025. Magen David Adom (MDA), the national emergency medical service, provides unified prehospital care for the region. The study followed STROBE reporting guidelines.

### Patient population

Adult patients (≥18 years) with refractory OHCA who were transported to SZMC under mechanical CPR and cannulated for ECMO during ongoing resuscitation were included. This represents an as-treated ECPR cohort; non-cannulated OHCA patients were generally not available in the dataset, although screening denominators were obtainable for a recent subperiod.

### Local ECPR workflow

ECPR candidacy was assessed using prehospital and early in hospital features associated with favorable outcomes after refractory cardiac arrest, including witnessed arrest with immediate bystander CPR, age up to 70 years old, without major comorbidities, physiological signs of ongoing perfusion during resuscitation, and an estimated low flow duration within approximately 60 min. EMS pre-notified the ICU of potential candidates, enabling multidisciplinary evaluation and rapid activation. Final decisions regarding cannulation were individualized, allowing clinical discretion in cases with intermittent return of spontaneous circulation or preserved physiological parameters.

### Post-ECPR management

After cannulation, patients underwent CT imaging and/or coronary angiography when indicated.^10^ Definitive interventions, including percutaneous coronary intervention (PCI) if appropriate, were performed prior to Intensive Care Unit (ICU) admission. A temperature below 36°C was maintained for 24 h, and fever was prevented for at least 72 h.^11^

### Data sources and variables

Data were obtained from EMS reports, ED documentation, electronic medical records, and ECMO logs. Extracted variables included demographics, comorbidities, arrest characteristics (witnessed status, no flow time, response intervals, initial rhythm, number of defibrillations, adrenaline dose), and ED laboratory values (lactate, pH, PaO_2_).

### Sample size

This study analyzed all the patients in whom ECPR was initiated at our institution. Therefore, no *a priori* sample calculations were performed.

### Ethics and funding

The SZMC Institutional Review Board approved the study (0281-23-SZMC) and waived informed consent. The study received internal support from the Eisenberg R&D Authority.

### Outcomes

The primary outcome was survival to hospital discharge among OHCA patients treated with ECPR, comparing patients who met versus did not meet inclusion criteria from the ARREST, PRAGUE, and INCEPTION trials. A key secondary outcome was hospital discharge with favorable neurological outcome, which was examined to align with the primary endpoints of the randomized trials but was not designated as a primary outcome due to the low event rate and limited statistical power.

### Comparison with randomized trials

To evaluate compatibility with RCT eligibility criteria, each patient was assessed against the inclusion thresholds used in ARREST, PRAGUE, and INCEPTION. Lactate values were capped at the analyzer’s upper reporting limit (15 mmol/L). Although ETCO_2_ values were available to clinicians during resuscitation and informed bedside decision-making, they were not reliably recorded in the medical record and therefore could not be incorporated into retrospective eligibility assessment. Two patients lacked complete prehospital time documentation and were excluded from time-dependent analyses. For the ARREST PaO_2_ requirement (≥50 mmHg), all patients without PaO_2_ measurements at ED arrival had already been excluded earlier in the analysis, so no additional exclusions were necessary for that criterion. For the INCEPTION comparison, call to hospital arrival up to 60 min, was used to approximate prospective ECMO eligibility, consistent with the trial’s observed cannulation delays, and robustness was assessed by varying the arrival-time threshold.

### Statistical analysis

Continuous variables were summarized as medians (IQR) and bootstrapped means (SD). Categorical variables were presented as counts and percentages. Comparisons with RCT cohorts used published summary statistics; when only medians and IQRs were available, means and SDs were approximated using established formulas.

Because several variables were non-normally distributed and the cohort size was small, bootstrap resampling was used to generate confidence intervals for continuous variables, and permutation tests were used for hypothesis testing without relying on distributional assumptions. For categorical comparisons, chi-square tests were applied when expected counts were adequate and Fisher’s exact tests when sparse data were present.

Confidence intervals for single proportions were calculated using the Clopper–Pearson exact method, chosen for its reliable coverage in small samples.

In addition to frequentist comparisons, survival was analyzed using a Bayesian Beta–Binomial framework to estimate posterior survival probabilities and credible intervals and to assess non-inferiority and clinical equivalence relative to randomized trial benchmarks.

Sensitivity analyses were performed by assigning missing outcomes under worst-case, best-case, and neutral assumptions across eligibility strata to evaluate the robustness of observed survival differences.

All analyses were conducted in R version 4.3.3 using the boot and coin packages, with a seed of 1234 set for reproducibility. A *p* value < 0.05 was considered significant.

### Handling of missing data

Missingness affected prehospital time intervals and select laboratory values. No imputation was performed. Detailed documentation of bystander CPR and AED use prior to EMS arrival was incomplete; therefore, initiation of CPR within 10 min of collapse (‘short now flow time’) was used as a surrogate marker of early resuscitation. For eligibility classification, missing values required for a specific criterion resulted in coding the patient as not meeting that criterion (taken out from the “met” criteria), which may introduce misclassification bias. However, we have done a sensitivity analysis as described.

### Bias and methodological considerations

Analyses were restricted to patients who underwent ECPR cannulation and therefore represent program level benchmarking of trial eligibility rather than population based estimates of external validity.

Information bias was possible due to incomplete prehospital documentation; EMS and hospital timestamps were cross-validated when discrepancies occurred. Measurement bias occurred when laboratory values exceeded analyzer ranges; these were recorded at the nearest measurable threshold. Because all analyses were performed on an as-treated ECPR cohort, selection bias is inherent, and results cannot be extrapolated to all OHCA patients. Small sample size and limited survivors increase the risk of type II error, particularly in subgroup comparisons (STROBE checklist – Supplementary Table 1).

## Results

### Cohort characteristics

Sixty-six patients underwent ECPR for OHCA during the study period. During an interval with complete screening data (July 2022–April 2025), the denominator of patients evaluated for potential ECPR was available (134 patients) and used for decline-rate calculations; 54 (40%, 95% CI 32–49%) underwent ECPR.

The mean age of the cohort was 50.5 ± 15 years, and 82% were male. Coronary disease was the presumed etiology in 65% of cases, and 60.6% presented with an initial shockable rhythm. Mean time to ALS arrival was 11 ± 10 min (*n* = 64); EMS-call-to-hospital arrival was 45 ± 20 min; and mean time from call to ECMO initiation was 73 ± 20 (*n* = 59) minutes (Table 1, Table 2, Table 3).

**Table 1:** Demographic comparison across ECPR studies. Baseline characteristics reported in the ARREST, PRAGUE, and INCEPTION trials compared with the Shaare Zedek Medical Center (SZMC) cohort. Continuous variables are presented as mean ± SD or median (IQR), according to each study’s reporting methodology.

**Parameter**	**ARREST**	**PRAGUE**	**INCEPTION**	**SZMC**	**95% CI (Mean)**	**95% CI (Median)**
*N*	15	124	70	66		
True ECMO, *n*	12	79	46	66		
Age, years – Mean ± SD	59 ± 10*‡		54 ± 12	50.5 ± 15	46.7–54.2	
Age, years – Median (IQR)		59 (48–66)		54 (43–62)		50–59
Male sex, *n* (%)	14 (93)	102 (82)	63 (90)	54 (82)	NA	NA
Respiratory disease, *n* (%)	2 (13)	8/105 (8)**	20/35 (57)	23 (35)	NA	NA
Congestive heart failure, *n* (%)	1 (7)	11/106 (10)	4/62 (6)	7 (11)	NA	NA
Ischemic heart disease, *n* (%)	2 (13)	17/104 (16)	17 (28)**	14 (21)	NA	NA
Hypertension, *n* (%)	2 (13)	47/108 (44)	22/44 (55)	27 (41)	NA	NA
Chronic renal failure, *n* (%)	0 (0)	3/104 (3)	NA	4 (6)	NA	NA
Diabetes mellitus, *n* (%)	3 (20)	19/104 (18)*	10 (16)**	23 (35)	NA	NA

95% confidence intervals apply to SZMC age only. *P*-values were calculated using chi-square or Fisher’s exact test for categorical variables or permutation testing of emulated data for continuous variables. NA = not applicable.

‡ Outside the calculated 95% confidence interval (CI).

* *p* < 0.05.

** *p* < 0.01.

**Table 2:** Prehospital variables. This table compares prehospital and resuscitation characteristics reported in the ARREST, PRAGUE, and INCEPTION trials with Shaare Zedek Medical Center (SZMC) data. Continuous variables are presented as mean ± SD and/or median (IQR), according to each study’s reporting methods.

**Parameter**	**ARREST**	**PRAGUE**	**INCEPTION**	**SZMC**	**95% CI Mean**	**95% CI Median**	**SZMC *n***
Call to EMS (min) – Mean ± SD	6 ± 2.3*‡		8 ± 4*‡	11 ± 10	9.00–13.9		64
Call to EMS (min) – Median (IQR)		8 (7–11)		9 (6–14)		7–13	64
Call to hospital (min) – Mean ± SD	48.5 ± 21**		36 ± 12**‡	45 ± 20	39.9–50.1		64
Call to hospital (min) – Median (IQR)		49 (44–60)**‡		42 (33–52)		37.5–46	64
Short no flow time, *n* (%)	11 (73)	123 (99)**	69 (99)**	38 (58)			66
Shockable rhythm, *n* (%)	15 (100)**	72 (58)	69 (99)**	40 (61)			66
Adrenaline (mg) – Mean ± SD	3.3 ± 2.3**‡		9 ± 4**‡	4.6 ± 2.5	4–5.17		66
Adrenaline (mg) – Median (IQR)		4 (2–5)		4 (3–7)		4–5	66
Defibrillations – Mean ± SD	5 ± 2.5		8 ± 5**‡	4.8 ± 5.2	3.61–6.06		66
Defibrillations – Median (IQR)		4 (2–6)		3 (1–9)		1–4	66
**Etiology of arrest, *n* (%)**							
Coronary disease	15 (100)**	78 (63)	51 (73)	43 (65)			66
Pulmonary embolism	0 (0)	12 (10)	1 (1)	3 (5)			66
Cardiomyopathy	0 (0)	17 (14)	13 (19)	6 (9)			66
Other	0 (0)	17 (14)	3 (4)**	14 (21)			66

95% confidence intervals apply to SZMC age only. *P*-values were calculated using chi-square or Fisher’s exact test for categorical variables or permutation of emulated data. EMS = emergency medical services; BLS = basic life support.

‡ Outside the calculated 95% confidence interval (CI).

* *p* < 0.05.

** *p* < 0.01.

**Table 3:** Hospital variables. Hospital characteristics reported in the ARREST, PRAGUE, and INCEPTION trials compared with the Shaare Zedek Medical Center (SZMC) cohort. Continuous variables are presented as mean ± SD or median (IQR), according to each study’s original reporting. Confidence intervals are shown only for SZMC-derived estimates.

**Parameter**	**ARREST**	**PRAGUE**	**INCEPTION**	**SZMC**	**95% CI (Mean, SZMC)**	**95% CI (Median, SZMC)**	**SZMC *n***
Initial pH – Mean ± SD	6.9 (0.9)		6.97 ± 0.16*‡	6.9 ± 0.1	6.85–6.90		66
Initial pH – Median (IQR)		6.93 (6.80–7.10)**‡		6.84 (6.80–6.93)		6.83–6.88	66
Initial lactate, mmol/L – Mean ± SD	11.5 (4.5)		13 ± 5	12 ± 3	10.9–12.4		66
Initial lactate, mmol/L – Median (IQR)		12.5 (9.2–16)		11.8 (9.2–15)		10.8–13.3	66
Initial O_2_, mmHg – Mean ± SD	86 (18)**			56 ± 25	50.3–63.3		60
Initial O_2_, mmHg – Median (IQR)			60 (22–135)	55 (46–67)		52–59.5	60
Time to ECMO run, min – Mean ± SD	59 ± 28‡			73 ± 20	68.2–78.3		59
Time to ECMO run, min – Median (IQR)		61 (55–70)**	74 (63–87)	70 (58–74)		64–77	59
Coronary disease, *n* (%)	15 (100)**	78 (63)	51 (73)	43 (65)			66
Pulmonary embolism, *n* (%)	0 (0)	12 (10)	1 (1)	3 (5)			66
Cardiomyopathy, *n* (%)	0 (0)	17 (14)	13 (19)	6 (9)			66
Other etiology, *n* (%)	0 (0)	17 (14)	3 (4)**	14 (21)			66
Cardiac catheterization, *n* (%)	13 (87)	120/124 (98)**	34 (49)*	47 (71)			66
Survival, *n* (%)	6 (50)	16 (22)	5 (11)	9 (14)			66
Survival 95% CI	21–79**	13–32	4–24	6–24			66
Time on ECMO, days – Mean ± SD			1.1 ± 1.8**‡	6 ± 11	3.79–9.52		66
Time on ECMO, days – Median (IQR)	4 (2–21)#			4 (2–6)		3–5	66

95% confidence intervals apply to SZMC age only. *P*-values were calculated using chi-square or Fisher’s exact test for categorical variables or permutation of emulated data. Survival proportion CIs were calculated using the Clopper–Pearson method.

‡ Outside the calculated 95% confidence interval (CI).

* *p* < 0.05.

** *p* < 0.01.

# Only for survivors.

Nine patients survived to hospital discharge (13.6%, 95% CI 6.4–24.3%). Among survivors with an initial shockable rhythm, 4 of 6 (67%) achieved Cerebral Performance Category (CPC) 1–2, and one additional patient was transferred awake on Impella 5.5 to another hospital. Among survivors with an initial non-shockable rhythm, 1 of 3 (33%) attained CPC 1–2. Additional characteristics appear in Table 2.

### Survival within the SZMC cohort by RCT inclusion criteria

**Fig. 1:**
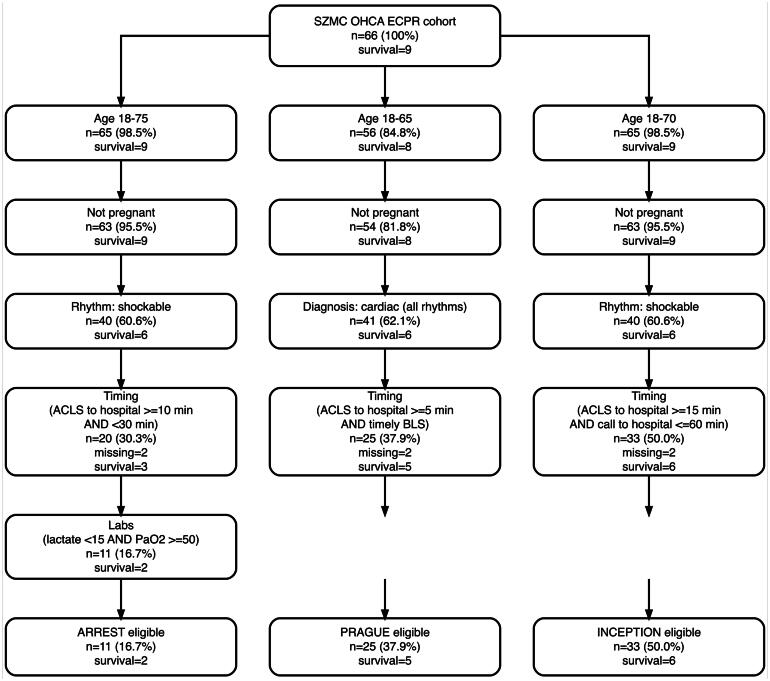
**Program-level application of ARREST, PRAGUE, and INCEPTION inclusion criteria among cannulated ECPR patients.** Inclusion criteria were applied exclusively to patients who underwent ECPR cannulation and therefore reflect program-level benchmarking rather than population-based eligibility. The figure illustrates the derivation of the Shaare Zedek Medical Center (SZMC) ECPR cohort and the sequential application of inclusion criteria from the ARREST, PRAGUE, and INCEPTION trials. Eligibility steps are organized into harmonized domains to facilitate visual comparison while preserving trial-specific logic. *Abbreviations:* OHCA, out-of-hospital cardiac arrest; ECPR, extracorporeal cardiopulmonary resuscitation; ACLS arrival, time of emergency medical services arrival on scene; timely BLS, initiation of basic life support within 10 min of collapse; PaO_2_, arterial partial pressure of oxygen; SZMC, Shaare Zedek Medical Center.

**Fig. 2:**
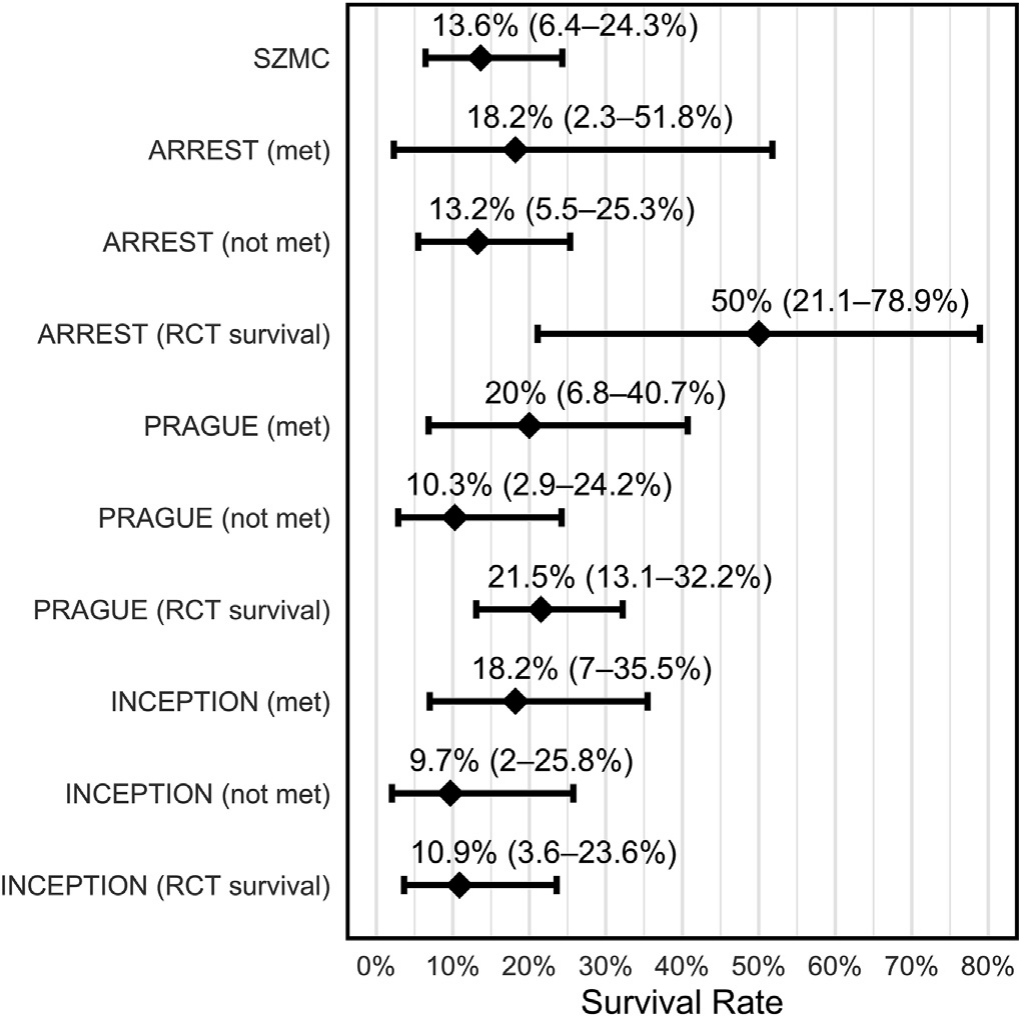
**Forest plot of confidence intervals – CI (using the binomial Clopper-Pearson method)**. CI of survival rates of SZMC – Shaare Zedek Medical Center and each of the randomized controlled trials (RCT’s) when their inclusion criteria were met or not met by the SZMC cohort. In addition, the survival rate of each RCT treatment arm was included.

When stratifying the cohort according to the inclusion criteria of ARREST, PRAGUE, and INCEPTION, Among patients with complete eligibility data (*n* = 64), survival was numerically higher among patients who met the respective criteria, but none of the differences reached statistical significance and all confidence intervals were wide (Fig. 1, Fig. 2). For ARREST, survival was 18.2% (2 of 11, 95% CI 2.3–51.8%) in patients meeting the criteria and 13.2% (7 of 53, 95% CI 5.5–25.3%) in those who did not (*p* = 0.65). For PRAGUE, survival was 20.0% (5 of 25, 95% CI 6.8–40.7%) versus 10.3% (4 of 39, 95% CI 2.9–24.2%) (*p* = 0.30). For INCEPTION, survival was 18.2% (6 of 33, 95% CI 7–35.5%) versus 9.7% (3 of 31, 95% CI 2–25.8%) (*p* = 0.48). These findings were consistent across sensitivity analyses of missing outcome classification, using worst case, best case, and neutral assumptions for missing outcomes (see supplementary Table 2).

### Neurological outcome within the SZMC cohort

When neurological outcome at hospital discharge was examined, no statistically significant differences in favorable neurological outcome (CPC 1–2) were observed either within the cohort (met vs not met trial eligibility) or between the overall SZMC cohort and patients meeting ARREST, PRAGUE, or INCEPTION criteria. Nevertheless, application of increasingly restrictive trial criteria was associated with progressively higher observed proportions of favorable neurological outcome, with the lowest proportion in the SZMC cohort and higher proportions observed sequentially for INCEPTION, PRAGUE, and ARREST (Supplementary Table 3 and Fig. 3), mirroring the pattern seen for survival to hospital discharge.

### Comparison with RCT populations (exploratory benchmarking)

Compared with ARREST, PRAGUE, and INCEPTION, the SZMC cohort differed across several demographic, clinical, and process related characteristics. SZMC patients were younger than those in ARREST and had higher rates of smoking, chronic lung disease, and diabetes than patients in PRAGUE, as well as higher diabetes prevalence but less known ischemic heart disease than INCEPTION. A smaller proportion presented with a shockable rhythm compared with ARREST and INCEPTION, and immediate life-support initiation was less frequent than in PRAGUE and INCEPTION.

Prehospital intervals were longer than those reported in ARREST and INCEPTION and intermediate relative to PRAGUE. Admission physiology was more deranged, while rates of cardiac catheterization were lower than in PRAGUE. As-treated ECMO survival was lower than in ARREST but broadly similar to PRAGUE and INCEPTION. These comparisons are presented as exploratory benchmarking rather than inferential contrasts.

### Comparison of SZMC survival with survival in RCTs

Among patients who were cannulated, survival at SZMC (13.6%, 95% CI 6.4–24.3%) was comparable to the survival observed in PRAGUE (20.3%, 95% CI 12.0–30.8%; *p* = 0.28) and INCEPTION (10.9%, 95% CI 3.6–23.6%; *p* = 0.78).

Survival in ARREST (50.0%, 95% CI 21.1–78.9%) was numerically higher; although the confidence intervals overlapped, Fisher’s exact test showed a statistically significant difference relative to the SZMC cohort (*p* = 0.009). Confidence intervals were wide in both studies; therefore, this finding should be interpreted with caution.

All between-study comparisons are summarized in Fig. 2.

### Bayesian analysis

Bayesian Beta–Binomial analyses showed that while intention to treat survival in randomized trials exceeded that observed at SZMC, posterior distributions among ECMO treated patients overlapped substantially for PRAGUE and INCEPTION, with moderate to high posterior probabilities of non inferiority, particularly for INCEPTION (Supplementary Table 4).

## Discussion

### Principal findings

This study evaluated outcomes and eligibility mapping within a single center ECPR program for OHCA, focusing on how commonly used randomized trial inclusion criteria classify patients and stratify outcomes in routine clinical practice. Overall survival to hospital discharge was low, but survival occurred among patients who did not meet RCT inclusion criteria, indicating that trial derived frameworks do not fully encompass all patients with potential for meaningful recovery.

Although the cohort was modest in size when compared to large registries, it is comparable to many single-center ECPR studies in the resuscitation literature, where enrollment is frequently limited by the rarity and complexity of the intervention. These considerations underscore the challenges of translating trial-based inclusion criteria into broader clinical environments.^12^, ^13^

### Eligibility criteria and implications for patient selection

Randomized trial inclusion criteria are designed to optimize internal validity and are often used as reference points for clinical decision-making and guideline development. Evaluating how these criteria map onto patients treated within our program provides insight into their practical applicability rather than a test of their performance.

Survival in refractory OHCA remains poor, and prior trials have reported conflicting results regarding the role of ECPR.^5^, ^14^ Still, in patients with refractory arrest unresponsive to conventional CPR, ECPR is generally accepted as a strategy that may reduce mortality,^5^ although in-hospital mortality remains high.^3^, ^14^ In our cohort, applying increasingly restrictive criteria substantially reduced the proportion of patients deemed eligible; however, we did not observe a robust statistically significant survival advantage among those meeting stricter definitions.

Our findings highlight two inherent tensions in ECPR practice: first, between providing a time-critical, last-resort therapy and responsible stewardship of a highly resource-intensive intervention; and second, between striving toward trial-defined inclusion standards and enforcing strict eligibility boundaries in dynamic clinical settings. These tensions suggest that trial criteria should be viewed as directional benchmarks that guide system evolution and patient selection boundaries, rather than as rigid rules applied without regard to operational realities.

### Comparison with outcomes from randomized trials

Comparisons with randomized trials should be interpreted as contextual benchmarking rather than direct statistical contrasts. Survival in our cohort was lower than intention-to-treat survival reported in ARREST, PRAGUE, and INCEPTION. However, interpretation must consider important methodological differences. INCEPTION and PRAGUE randomized patients prehospital, and many achieved ROSC before hospital arrival, never undergoing cannulation. ARREST enrolled fewer, highly selected patients, which may explain its superior outcomes. In contrast, our cohort included only those who failed ROSC, making it more comparable to the as-treated populations in the RCT intervention arms. Under this framework, survival among patients meeting RCT inclusion criteria in our study broadly overlapped with that reported in PRAGUE and INCEPTION, although lower than ARREST. These findings emphasize the trade-off between efficiency gained through strict criteria and inclusivity offered by broader eligibility, and suggest that meaningful benefit may still be possible even when patients fall outside trial-defined thresholds.^15^

### Non-shockable rhythms and expanding eligibility

A key distinction between our cohort and the RCTs was the inclusion of patients with non-shockable rhythms. Although shockable rhythms are consistently associated with better outcomes across combined trial datasets,^16^, ^17^ we observed survival with favorable neurological outcomes in a minority of patients presenting with non-shockable rhythms. These results are consistent with registry data. A Japanese study demonstrated a 5.37-fold survival benefit with ECPR for non-shockable OHCA compared with conventional management.^18^ Similarly, a Danish registry reported that although asystole carries high mortality, neurologically intact survivors do occur, particularly among patients with pulseless electrical activity (PEA), signs of life during CPR, and shorter low flow times.^19^ Together, these findings suggest that non-shockable rhythm alone should not be considered an absolute exclusion criterion in all settings.

### System-level factors and their influence on outcomes

Differences between trial settings and our program likely limit the discriminatory performance of RCT inclusion criteria when applied outside their original contexts. System-level factors known to influence ECPR outcomes—including EMS response times, activation pathways, and cannulation logistics—vary substantially across programs.^1^, ^20^ ARREST and PRAGUE were conducted in single, highly experienced centers with streamlined workflows and shorter low flow intervals than observed in our system, whereas INCEPTION involved heterogeneous centers with variable low flow times.^6^, ^7^, ^8^ Differences in prehospital activation strategies and cannulation location may further contribute to these observations. In addition, the SZMC cohort had higher rates of smoking, chronic respiratory disease, and diabetes, reflecting a greater burden of baseline comorbidity, and arrest etiology was more heterogeneous—both factors known to influence survival probability.^21^, ^22^, ^23^

### Limitations

As a single center analysis restricted to patients who underwent ECPR cannulation, the findings reflect program-level experience and cannot be generalized to the broader OHCA population. Comparisons with randomized trials relied on published summary statistics rather than individual patient data, requiring assumptions and limiting precision.

The study period overlapped with publication of the RCTs; although trial-specific criteria were not formally adopted, indirect influence on clinical decision-making cannot be excluded. In addition, detailed Utstein-style cardiac arrest characteristics (e.g., bystander CPR, AED use, witnessed status) were incompletely available, limiting adjustment for arrest severity and interpretation of survival differences within the ECPR cohort.

Our as-treated analytic approach differs from intention-to-treat analyses used in RCTs and may either underestimate or overestimate treatment effects. The modest sample size and low event rate increase the risk of type II error. Several trial-specific variables were incompletely available, and the ECPR program evolved over the study period, including during the COVID-19 pandemic and regional conflict, which may have influenced operational performance.

## Conclusions

Applying RCT derived inclusion criteria for ECPR in OHCA may exclude some patients with the potential for meaningful recovery. While such criteria are helpful for structuring care pathways and guiding resource use, they do not translate uniformly across different health systems or operational contexts. Our findings highlight the need to refine patient selection strategies and to account for system-level capabilities when implementing ECPR programs. Until stronger evidence is available, clinical judgment remains essential in guiding ECPR decision-making.

## CRediT authorship contribution statement

**Yigal Helviz:** Writing – review & editing, Writing – original draft, Validation, Methodology, Funding acquisition, Formal analysis. **Frederic Shmuel Zimmerman:** Writing – review & editing, Writing – original draft, Visualization. **Amir Orlev:** Writing – original draft, Visualization, Validation. **Michael Glikson:** Validation, Conceptualization. **David Shimony:** Resources, Investigation. **Daniel Fink:** Validation, Resources, Investigation. **Refael Strugo:** Validation, Resources, Investigation. **Elad Asher:** Validation, Resources. **Tomer Kaplan:** Validation. **Reem Naimy:** Resources, Investigation. **Ofer Merin:** Validation, Resources. **Phillip D. Levin:** Validation, Supervision.

## Funding

This study was supported by The Eisenberg R&D Authority, Shaare Zedek Medical Center. The funds were used for the data analysis.

## Declaration of competing interest

The authors declare that they have no known competing financial interests or personal relationships that could have influenced the work reported in this study.
