# Supplementary material for: Applying randomized control trial criteria to an ECPR cohort
Source: Resusc Plus. 2026 Feb 12;28:101269. doi: 10.1016/j.resplu.2026.101269 (PMC13080480; doi:10.1016/j.resplu.2026.101269)
Supplement: Supplementary Table 1 [file mmc1.docx]

# STROBE Checklist for Cohort Studies

Manuscript title: ECPR for OHCA: Applicability of Randomized Trial Inclusion Criteria in a Single-Centre Cohort

| Section/Topic | Item | Recommendation | Addressed in Manuscript |
| --- | --- | --- | --- |
| Title & Abstract | 1a | Indicate study design with commonly used terms | Title, Abstract |
| Title & Abstract | 1b | Informative and balanced abstract | Abstract |
| Introduction | 2 | Background/rationale | Introduction |
| Introduction | 3 | Objectives/hypotheses | End of Introduction |
| Methods | 4 | Key elements of study design | Methods — First paragraph |
| Methods | 5 | Setting, locations, dates | Methods — Setting and Patients; Figure 1 |
| Methods | 6a | Eligibility criteria and cohort assembly | Methods — Patients; Application of RCT criteria |
| Methods | 6b | Matching criteria | Not applicable |
| Methods | 7 | Define outcomes, exposures, confounders | Methods — Outcomes |
| Methods | 8 | Source of data and assessment | Methods — Data Collection |
| Methods | 9 | Efforts to address bias | Methods — Bias; Discussion — Limitations |
| Methods | 10 | Sample size rationale | Methods — Sample Size Considerations |
| Methods | 11 | Handling quantitative variables | Methods — Statistical Analysis |
| Methods | 12a | Statistical methods | Methods — Statistical Analysis |
| Methods | 12b | Subgroup/interaction analyses | Methods — Stratified Analyses |
| Methods | 12c | Missing data handling | Methods — Missing Data |
| Methods | 12d | Loss to follow-up | Not applicable |
| Methods | 12e | Sensitivity analyses | Methods — Permutation & Bootstrap Analyses |
| Results | 13a | Numbers at each stage | Results — Cohort description; Figure 1 |
| Results | 13b | Reasons for non-participation | Results — Flow/Limitations |
| Results | 13c | Flow diagram | Figure 1 |
| Results | 14a | Participant characteristics | Table 1; Results |
| Results | 14b | Missing data by variable | Tables 1–4; Supplement |
| Results | 15 | Outcome events | Results; Tables 2–4 |
| Results | 16a | Estimates with precision | Results — Comparative Analyses; Table 3 |
| Results | 16b | Category boundaries | Tables 2–4 |
| Results | 16c | Translate RR to absolute risk | Not applicable |
| Results | 17 | Other analyses | Results — Stratified analyses |
| Discussion | 18 | Key results summary | Discussion — Opening |
| Discussion | 19 | Limitations | Discussion — Limitations |
| Discussion | 20 | Overall interpretation | Discussion — Final |
| Discussion | 21 | Generalizability | Discussion — Applicability paragraph |
| Other Information | 22 | Funding source/role | Funding Statement |

This study adhered to the STROBE guidelines. A completed checklist is provided above.
