# Supplementary material for: Applying randomized control trial criteria to an ECPR cohort
Source: Resusc Plus. 2026 Feb 12;28:101269. doi: 10.1016/j.resplu.2026.101269 (PMC13080480; doi:10.1016/j.resplu.2026.101269)
Supplement: Supplementary Table 2 [file mmc2.docx]

# Supplementary Table 2. Sensitivity Analyses of Survival by Trial Eligibility

Sensitivity analyses examining survival to hospital discharge among patients meeting versus not meeting INCEPTION and PRAGUE trial inclusion criteria under alternative assumptions regarding missing outcome data. Results are shown as number of survivors/total patients (%). Fisher’s exact p-values reflect exact two-sided comparisons between patients meeting versus not meeting trial criteria. Comparisons are exploratory.

| Trial | Baseline assumption | Scenario | Met | Not met | Risk difference | Risk ratio | Exact Fisher p (Met vs Not met) |
| --- | --- | --- | --- | --- | --- | --- | --- |
| INCEPTION | 64 | +1 death to MET & +1 to NOT MET | 6/34 (17.6%) | 3/32 (9.4%) | 8.3% | 1.88 | 0.48 |
| INCEPTION | 64 | +2 deaths to MET | 6/35 (17.1%) | 3/31 (9.7%) | 7.5% | 1.77 | 0.48 |
| INCEPTION | 64 | +2 deaths to NOT MET | 6/33 (18.2%) | 3/33 (9.1%) | 9.1% | 2.00 | 0.48 |
| INCEPTION | 64 | Baseline | 6/33 (18.2%) | 3/31 (9.7%) | 8.5% | 1.88 | 0.48 |
| INCEPTION | 65 | +1 death to MET | 6/34 (17.6%) | 3/32 (9.4%) | 8.3% | 1.88 | 0.48 |
| INCEPTION | 65 | +1 death to NOT MET | 6/33 (18.2%) | 3/33 (9.1%) | 9.1% | 2.00 | 0.48 |
| INCEPTION | 65 | Baseline | 6/33 (18.2%) | 3/32 (9.4%) | 8.8% | 1.94 | 0.48 |
| PRAGUE | 64 | +1 death to MET & +1 to NOT MET | 5/26 (19.2%) | 4/40 (10.0%) | 9.2% | 1.92 | 0.30 |
| PRAGUE | 64 | +2 deaths to MET | 5/27 (18.5%) | 4/39 (10.3%) | 8.3% | 1.81 | 0.47 |
| PRAGUE | 64 | +2 deaths to NOT MET | 5/25 (20.0%) | 4/41 (9.8%) | 10.2% | 2.05 | 0.28 |
| PRAGUE | 64 | Baseline | 5/25 (20.0%) | 4/39 (10.3%) | 9.7% | 1.95 | 0.30 |
| PRAGUE | 65 | +1 death to MET | 5/26 (19.2%) | 4/40 (10.0%) | 9.2% | 1.92 | 0.30 |
| PRAGUE | 65 | +1 death to NOT MET | 5/25 (20.0%) | 4/41 (9.8%) | 10.2% | 2.05 | 0.28 |
| PRAGUE | 65 | Baseline | 5/25 (20.0%) | 4/40 (10.0%) | 10.0% | 2.00 | 0.29 |
