# Supplementary material for: Applying randomized control trial criteria to an ECPR cohort
Source: Resusc Plus. 2026 Feb 12;28:101269. doi: 10.1016/j.resplu.2026.101269 (PMC13080480; doi:10.1016/j.resplu.2026.101269)
Supplement: Supplementary Table 3 [file mmc3.docx]

# Supplementary Table 3. Favorable neurological outcome (CPC 1–2)

A. Met vs not met eligibility within the SZMC cohort

| Trial criteria | Met: CPC 1–2 | Met: CPC 3–5 | Not met: CPC 1–2 | Not met: CPC 3–5 | Fisher p |
| --- | --- | --- | --- | --- | --- |
| ARREST | 2 | 9 | 3 | 50 | 0.20 |
| PRAGUE | 4 | 21 | 1 | 38 | 0.07 |
| INCEPTION | 4 | 29 | 1 | 30 | 0.36 |

B. Overall SZMC cohort vs patients meeting RCT eligibility criteria

| Comparison | CPC 1–2 / Total (SZMC) | CPC 1–2 / Total (RCT met) | Fisher p | Poisson rate ratio | Poisson p |
| --- | --- | --- | --- | --- | --- |
| SZMC vs INCEPTION | 5 / 66 (8%) | 4 / 33 (12%) | 0.48 | 1.6 | 0.49 |
| SZMC vs PRAGUE | 5 / 66 (8%) | 4 / 25 (16%) | 0.25 | 2.1 | 0.27 |
| SZMC vs ARREST | 5 / 66 (8%) | 2 / 11 (18%) | 0.26 | 2.4 | 0.26 |

CPC, Cerebral Performance Category. CPC 1–2 indicates favorable neurological outcome; CPC 3–5 indicates unfavorable neurological outcome or death. Neurological outcome was assessed at hospital discharge. Fisher exact tests were used for categorical comparisons due to small sample sizes. Poisson rate ratios are provided as supportive analyses.


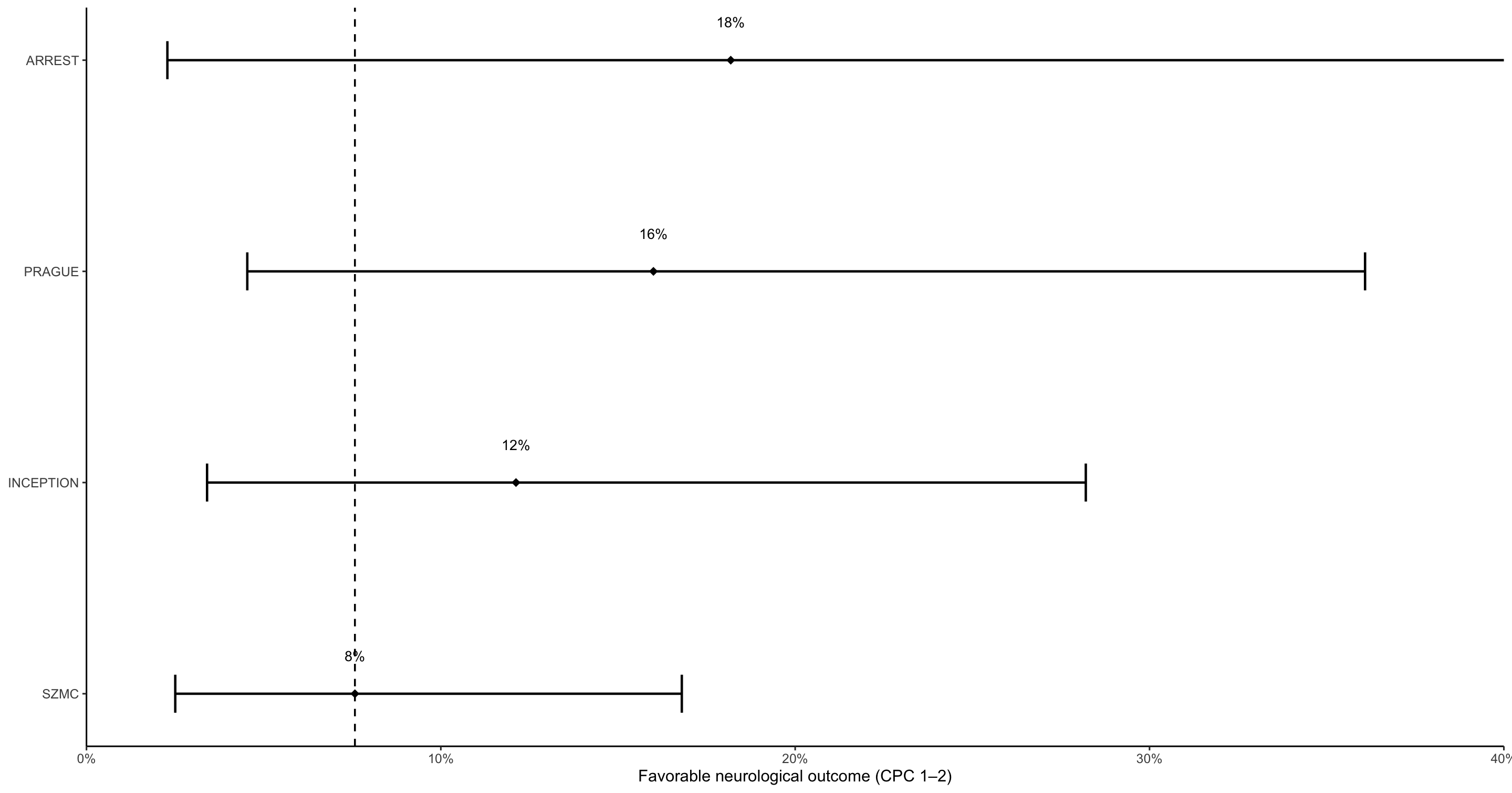


Supplementary Figure 3. Favorable neurological outcome according to trial eligibility criteria.

Forest plot showing the proportion of patients with favorable neurological outcome (CPC 1–2) at hospital discharge in the overall SZMC cohort and among patients meeting eligibility criteria for the ARREST, PRAGUE, and INCEPTION trials. Points represent observed proportions, horizontal bars indicate exact 95% confidence intervals, and percentages denote point estimates. The dashed vertical line represents the proportion observed in the SZMC cohort.
