# Supplementary material for: Applying randomized control trial criteria to an ECPR cohort
Source: Resusc Plus. 2026 Feb 12;28:101269. doi: 10.1016/j.resplu.2026.101269 (PMC13080480; doi:10.1016/j.resplu.2026.101269)
Supplement: Supplementary Table 4 [file mmc4.docx]

# Supplementary Bayesian Tables

## Supplementary Table 4a. Observed and posterior survival

| **Level** | **Study** | **Observed (y/n)** | **Posterior mean (95% CrI)** |
| --- | --- | --- | --- |
| Presumed ITT survival | SZMC | 9/66 | 0.147 (0.074–0.240) |
| Presumed ITT survival | ARREST_ITT | 6/14 | 0.438 (0.213–0.677) |
| Presumed ITT survival | PRAGUE_ITT | 40/124 | 0.325 (0.247–0.409) |
| Presumed ITT survival | INCEPTION_ITT | 14/70 | 0.208 (0.123–0.309) |
| Presumed ITT survival | POOLED_ITT | 60/208 | 0.290 (0.231–0.354) |
| Actual ECMO-run survival | SZMC | 9/66 | 0.147 (0.074–0.240) |
| Actual ECMO-run survival | ARREST_actualECMO | 6/12 | 0.500 (0.251–0.749) |
| Actual ECMO-run survival | PRAGUE_actualECMO | 17/79 | 0.222 (0.139–0.318) |
| Actual ECMO-run survival | INCEPTION_actualECMO | 5/46 | 0.125 (0.048–0.231) |
| Actual ECMO-run survival | POOLED_actualECMO | 28/137 | 0.209 (0.145–0.280) |

## Supplementary Table 4b. Pairwise Bayesian comparisons (rstan)

| **Level** | **Comparator** | **Ratio (95% CrI)** | **P(RCT > SZMC)** | **ROPE ±5%** | **NI ±5%** | **ROPE ±10%** | **NI ±10%** |
| --- | --- | --- | --- | --- | --- | --- | --- |
| Presumed ITT survival | ARREST_ITT | 0.337 (0.148–0.79) | 0.991 | 0.025 | 0.026 | 0.067 | 0.067 |
| Presumed ITT survival | PRAGUE_ITT | 0.444 (0.219–0.79) | 0.998 | 0.017 | 0.017 | 0.100 | 0.100 |
| Presumed ITT survival | INCEPTION_ITT | 0.698 (0.322–1.44) | 0.827 | 0.384 | 0.428 | 0.725 | 0.732 |
| Presumed ITT survival | POOLED_ITT | 0.498 (0.246–0.82) | 0.994 | 0.048 | 0.049 | 0.207 | 0.207 |
| Actual ECMO-run survival | ARREST_actualECMO | 0.291 (0.131–0.67) | 0.997 | 0.010 | 0.011 | 0.031 | 0.032 |
| Actual ECMO-run survival | PRAGUE_actualECMO | 0.656 (0.304–1.30) | 0.883 | 0.327 | 0.351 | 0.652 | 0.655 |
| Actual ECMO-run survival | INCEPTION_actualECMO | 1.210 (0.470–3.49) | 0.348 | 0.531 | 0.872 | 0.857 | 0.967 |
| Actual ECMO-run survival | POOLED_actualECMO | 0.694 (0.329–1.20) | 0.867 | 0.380 | 0.404 | 0.747 | 0.749 |

Legend: Prior Beta(1,1). Posterior survival is reported as mean (95% credible interval, CrI). Ratio is the posterior median of p(SZMC)/p(comparator). P(RCT > SZMC) is the posterior probability that p(comparator) > p(SZMC). ROPE (Region of Practical Equivalence) is P(|pSZMC−pRCT|≤δ) for δ=0.05 or 0.10. NI (non-inferiority) is P(pSZMC ≥ pRCT−δ). Pooled rows pool event counts across ARREST, PRAGUE, and INCEPTION within the specified level. ITT – Intension To Treat. Actual ECMO – Actual data of patient who were eventually connected to ECMO.
